# Different responses of Japanese encephalitis to weather variables among eight climate subtypes in Gansu, China, 2005–2019

**DOI:** 10.1186/s12879-023-08074-6

**Published:** 2023-02-23

**Authors:** Ruifen Li, Xiaohong Zhao, Yu Tian, Yanjun Shi, Xueyan Gu, Shuang Wang, Rui Zhang, Jing An, Li Su, Xuxia Wang

**Affiliations:** 1 Gansu Provincial Center for Disease Prevention and Control, Institute of Health Education, Lanzhou, 730000 China; 2 School of Public Health, Lanzhou University, Lanzhou, 730000 China; 3 School of Public Health, Institute of Maternal, Child and Adolescent Health, Lanzhou University, Lanzhou, 730000 China; 4 Gansu Provincial Center for Disease Prevention and Control, Institute of Immunization Program, Lanzhou, 730000 China

**Keywords:** Japanese encephalitis, CART, Köppen–Geiger, Climate conditions

## Abstract

This study evaluated epidemic temporal aspects of Japanese encephalitis (JE) and investigated the weather threshold of JE response across eight climate subtypes between 2005 and 2019 in Gansu Province, China. Epidemiological data were collected from the China Information System for Disease Control and Prevention (CISDCP). Three epidemic temporal indices [frequency index (*α*), duration index (*β*), and intensity index (*γ*)] were adopted for the comparison of epidemic features among different climate subtypes. In addition, the local indicators of spatial association (LISA) technique was used to detect the hot-spot areas. The category and regression tree (CART) model was used to detect the response threshold of weather variables in hot-spot areas across climate subtypes. Among eight climate subtypes in Gansu, in most hot-spot areas (i.e., high–high clusters), *α*, *β*, and *γ* were detected in the climate subtypes of subtropical winter dry (Cwa), temperate oceanic continental (Cwb), and continental winter dry (Dwa and Dwb). According to the CART analysis, a minimum monthly temperature is required for Japanese encephalitis virus (JEV) transmission, with different threshold values among the climatic subtypes. In temperate climate zones (Cwa and Cwb), this threshold is 19 °C at a 1-month lag. It is lower in continental winter dry climate zones: 18 °C in Dwa (snow climate, dry winter, and hot summer) and 16 °C in Dwb (snow climate, dry winter, and warm summer). Additionally, some areas of the areas with temperate arid (BWk and BSk) had the first JE cases. Further studies to detect whether the climate change influence the JEV’s distribution in Gansu Province are needed.

**Supplementary Information:**

The online version contains supplementary material available at 10.1186/s12879-023-08074-6.

## Introduction

Japanese encephalitis (JE) is an important public health concern that caused an estimated 100,308 global clinical cases in 2015 [1]. Its case-fatality rate is as high as 30–40%, and approximately 30–50% of those who survive JE may experience severe neurological and mental sequelae [2, 3]. However, there is no specific antiviral treatment for Japanese encephalitis virus (JEV) [4, 5]. Therefore, detecting the transmission character of JEV and proposing more target preventive methods are essential to prevent the outbreak of JE.

JE is caused by JEV, which is a vector-borne zoonotic flavivirus mainly transmitted through the zoonotic cycle of *Culex tritaeniorhynchus* mosquitoes [6]. *Culex tritaeniorhynchus* is the main vector in Gansu. The vector density and non-human-host densities (pigs/poultry) determine the rates of JEV transmission [7, 8]. Because vector density may be influenced by precipitation and temperature, climatic factors are drivers of JEV transmission dynamics [7, 9, 10]. The temperature and precipitation influence JEV transmission to humans by affecting the mosquito life cycle, virus ecology, and human behavior (thus, virus–human interaction) [11, 12]. The extrinsic incubation time of JE in the vector and the intrinsic incubation time in the human host influence the time lags between weather conditions and incidence of JE. Therefore, because weather conditions may change the locations that are suitable for JEV transmission [12], probing these determined weather conditions in a certain area is vital.

Although Gansu is considered to be a low JE epidemic region [13], it was the top-ranking region for JE cases in China in 2017–2018 [14]. JE resulted in 945.85 potential years of life lost (PYLLs) in 2017 and 1056.92 PYLLs in 2018, causing a significant cost to families and society [15]. It remains unclear, however, which areas are at high risk for JEV transmission and whether weather factors have contributed to the observed variations in JE distribution over the past decade.

JE incidence rates respond to weather thresholds, which may vary in different areas. For example, the 22.8–34.5 °C and 21.0–25.2 °C temperatures in India [16] and Jinan (temperate climate with dry winters and wet, hot summers), China [17], respectively, were optimal for JEV transmission. Because JEV transmission dynamics are sensitive to weather conditions (temperature and rainfall), and the weather conditions vary in different climate subtypes, specifying the epidemic pattern in different climate zones is important for the accurate prevention of JE incidence in Gansu and similar climate zones. The climate classification is a general description of an area. Therefore, analyzing the epidemic pattern and relation between climate conditions and the incidence of JE based on the climate subtypes helps construct targeted prevention methods. However, no study has investigated the JE epidemic pattern in Gansu based on climate classification nor the response threshold of JE in different climate subtypes. Therefore, our study aims to construct a region-specific prediction model in high-risk areas in Gansu through category and regression tree (CART), which may help reveal the relationship between climate variables and the incidence of JE in different climate subtypes in Gansu.

## Materials and methods

### Study area and climate classification

Gansu Province is located in northwest China with a latitude between 32° 11′ N and 42° 57′ N and a longitude between 92° 13′ E and 108° 46′ E [14], covering an area of about 4.56 × 10^5^ km^2^ with 1383 towns (Additional file 1: Fig. S1). The population in 2019 was about 26.3 million in this area. Gansu Province Map information was obtained from the Chinese Centers for Disease Control (version 2012). The Köppen–Geiger climate classification map assigns 31 classifications incorporating three dimensions of climate conditions: the main climate, precipitation, and temperature [18–20]. According to the Köppen–Geiger climate classification, Gansu has eight different subtypes of climatic zones based on four major categories (B means arid, C means temperate, D means continental, and E means polar) [19]. Those climate subtypes, namely temperate arid (BWk), temperate semi-arid (BSk), subtropical winter dry (Cwa), temperate oceanic continental (Cwb), continental winter dry (Dwa: Snow climate, dry winter, and hot summer; and Dwb: Snow climate, dry winter, and warm summer), subpolar winter dry (Dwc), and alpine climate (ET), are marked in the map (Additional file 1: Fig. S1). Among these climate subtypes, the vegetation types are different: BWk is associated with deserts, BSk with steppes, Cwa and Cwb with temperate broadleaved evergreen forests, Dwa with temperate deciduous forest, Dwb with temperate/boreal mixed forest, Dwc with boreal deciduous forest/woodland, and ET with alpine tundra/polar desert [21].

### Data collection and aggregate units

#### Epidemiological data

JE is categorized as a class B notifiable infectious disease, and all levels of medical professionals are required to report JE infections through the China Information System for Disease Control and Prevention (CISDCP) [22, 23]. In our study, JE surveillance data from January 1, 2005 to December 31, 2019 included a total of 1528 cases obtained from the Notifiable Infectious Diseases Reporting Information System (NIDRIS). The clinical cases and laboratory-confirmed cases were included, and nine cases were excluded due to unclear location. The patient’s identifiers and precise location were removed to protect privacy. Cases were reported daily, and we aggregated cases weekly. To meet the demand for mapping, cases were summarized by town.

#### Meteorological data

Meteorological elements in Gansu Province were obtained from the Resource and Environment Science and Data Center of the Chinese Academy of Sciences (https://www.resdc.cn/Default.aspx). First, daily maximum temperature, daily minimum temperature, daily average temperature, and daily precipitation were collected from 72 meteorological stations in Gansu Province (Additional file 1: Fig. S1). Next, the study used the daily minimum temperature, maximum temperature, average temperature, and precipitation at 72 meteorological monitoring stations from 2005 to 2019 to calculate the monthly minimum, maximum, average temperature, and monthly rainfall at each meteorological monitoring station. Then, the ArcGIS 9.2 software was used to map rasterize layers of monthly climate elements from 2005 to 2019 using the ordinary Kriging method based on the town of Gansu Province. Finally, the average monthly minimum temperature, average monthly maximum temperature, average monthly temperature, and monthly township rainfall from 2005 to 2019 were extracted using the map.

The annual county population data were retrieved from the Health Statistical Yearbook of Gansu for 2005–2019, and LandScan™ was used to obtain the annual percentage of the township population. The annual proportion of the township population was multiplied by the annual county population to obtain the township population. The Institutional Review Board approved our study at the Gansu Provincial Center for Disease Control and Prevention (protocol code: 2020011). Informed consent was not required for this study because we aggregated the data without personal information.

### Epidemic temporal characteristics analysis

A JE epidemic can be described as either a sporadic or concentrated event. Although the incidence rate cannot show the time feature of JE, a sporadic JE epidemic may have the same incidence rate with concentrated onset. Because this is insufficient to evaluate the change patterns between epidemics in a timely manner, we used epidemic temporal features to characterize the JE epidemic pattern [24]. Three widely used temporal indices [frequency index (*α*), duration index (*β*), and intensity index (*γ*)] for modeling infectious diseases like dengue, influenza, and West Nile virus were adopted to describe the epidemic, which could be used to evaluate the severity and magnitude of epidemic risk [24]. The frequency index (*α*) is defined as follows:

$$\alpha = \frac{EW}{TW},$$

where EW or epidemic weeks means the total number of weeks in which one or more cases are notified, and TW or total weeks means the total number of weeks in the study period. This index ranges from 0 to 1, and as α approaches 1, the probability of JE cases occurring in certain weeks increases.

Because frequency index cannot help us identify the persistent disease period [24], duration index (*β*) is indispensable. This index is defined as follows:

$$\beta = \frac{EW}{EV},$$

where EW is as noted, and EV is the total epidemic waves during the whole study period. Epidemic waves are defined as the number of weeks when cases successively occur. Therefore, the duration index (*β*) refers to the average number of weeks in one JE epidemic wave, and *β* is important because it can evaluate the effectiveness of disease control strategies during an epidemic [24].

The incidence rate may reflect the disease burden in a specific period, but it cannot measure the magnitude of an epidemic. To describe the magnitude of a JE epidemic, an intensity index (*γ*) was defined. It has been formulated as follows:

$$\gamma = \frac{IR}{EV},$$

where the IR is the weekly incidence rate (cases per 100,000 population) during a JE epidemic period, and EV is as described above. A greater intensity index (*γ*) value implies a more temporally concentrated JE epidemic.

### Spatial pattern of JE epidemic indices

The temporal indices indicate the epidemic pattern, but the epidemic may be spatially heterogeneous [24]. Therefore, we identified the climate zones of hot-spot areas to discover the high-risk climate zones. Hence, global Moran’s *I* was used to examine whether the pattern is clustered, dispersed, or random [25]. Local indicators of spatial association (LISA) were used to detect spatial clusters of JE incidence at the town level between 2005 and 2019 [26]. The LISA cluster maps show the spatial clusters between a location's value and the mean of its neighboring values in the vicinity. The importance of the spatial correlations was evaluated using the Z-score. Although a low negative Z-score denoted a significant (*p*-value < 0.05) spatial outlier, a high positive Z-score showed that the surrounding features had either similarly high values (high–high) or similarly low values (low–low) (high–low or low–high). Spatial associations were performed using GeoDa™ (version 1.12) software.

### Interaction of weather and JE cases in different climate subtypes

Univariate linear regression analyses of town-level weather variables and the JE incidence rate in Gansu were performed to evaluate the most correlated lags of weather variables in different climate subtypes. To improve the fitting effects of patterns, we first performed a square root conversion of the monthly incidence. The weather variable had a maximum regression coefficient (absolute value), which was included in the model. CART is a flexible, robust, and non-parametric method that helps statisticians construct prediction models from continuous or categorical data [27]. It can be used for interactive exploration and description, prediction of patterns and processes [28], and dealing with multicollinear data [29]. The split feature is important in developing a tree, an algorithm that evaluates all of the possible splits to determine which circumstance explains the most variance in our indicator [30].

According to the epidemic analysis, areas with a high risk of JE incidence were selected to construct the CART models. The CARTs were constructed using monthly JE incidence as the response variable and the most correlated lag of weather variables as the independent variable. All analyses were conducted using R software (4.0.1), and the *rpart package* was used for CART.

## Results

### Temporal trends and seasonal pattern among eight climate subtypes

**Fig. 1:**
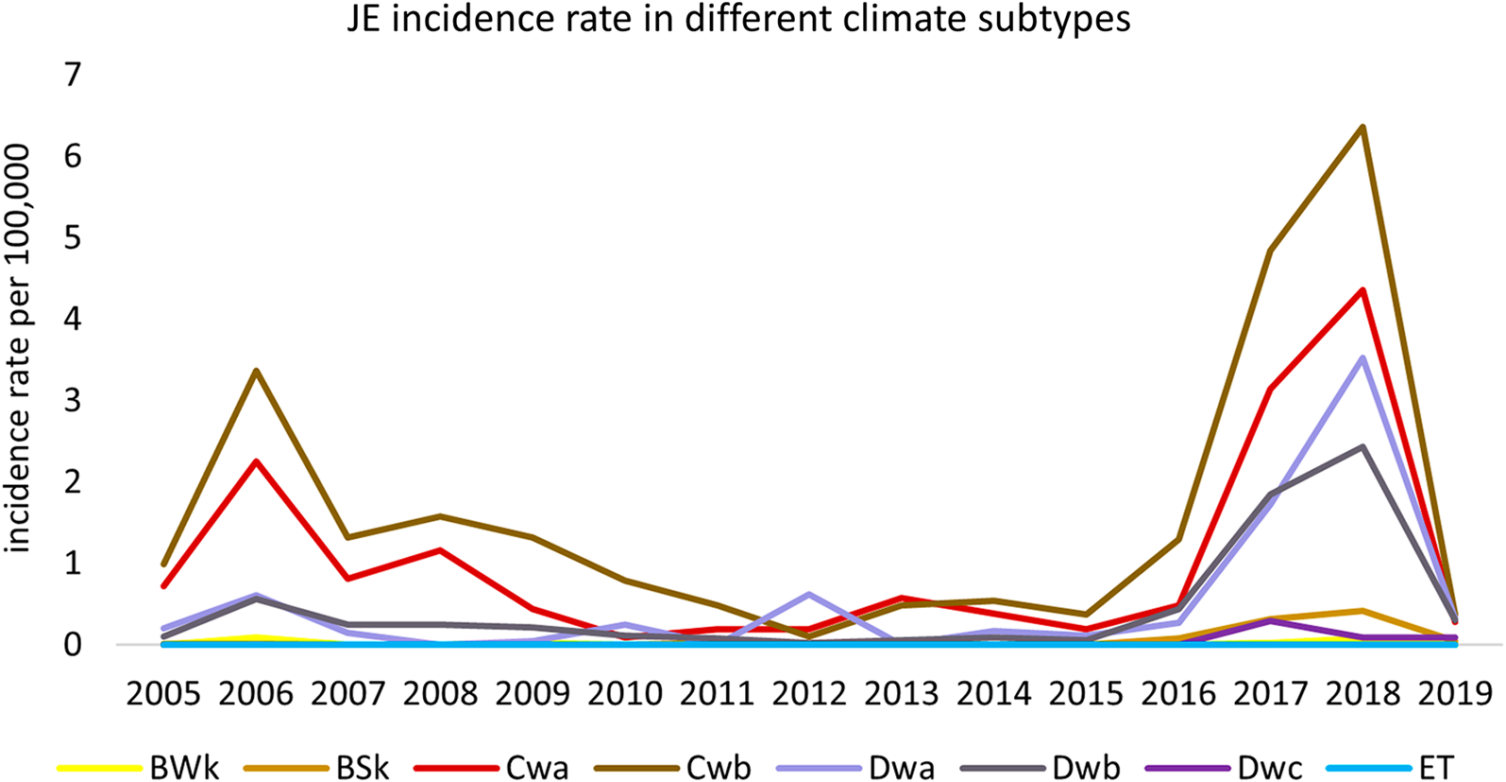
Incidence rate of JE in different climate subtypes in Gansu, 2015–2019. The Y-axis indicates the incidence rate (per 100,000), the x-axis means the year. Climate types: *BWk* temperate arid, *BSk* temperate semi-arid, *Cwa* subtropical winter dry, *Cwb* temperate oceanic continental, *Dwa and Dwb* continental winter dry, Dwa was characterized by snow climate, dry winter, and hot summer, Dwb was characterized by snow climate, dry winter, and warm summer, *Dwc* subpolar winter dry, *ET* alpine climate

**Fig. 2:**
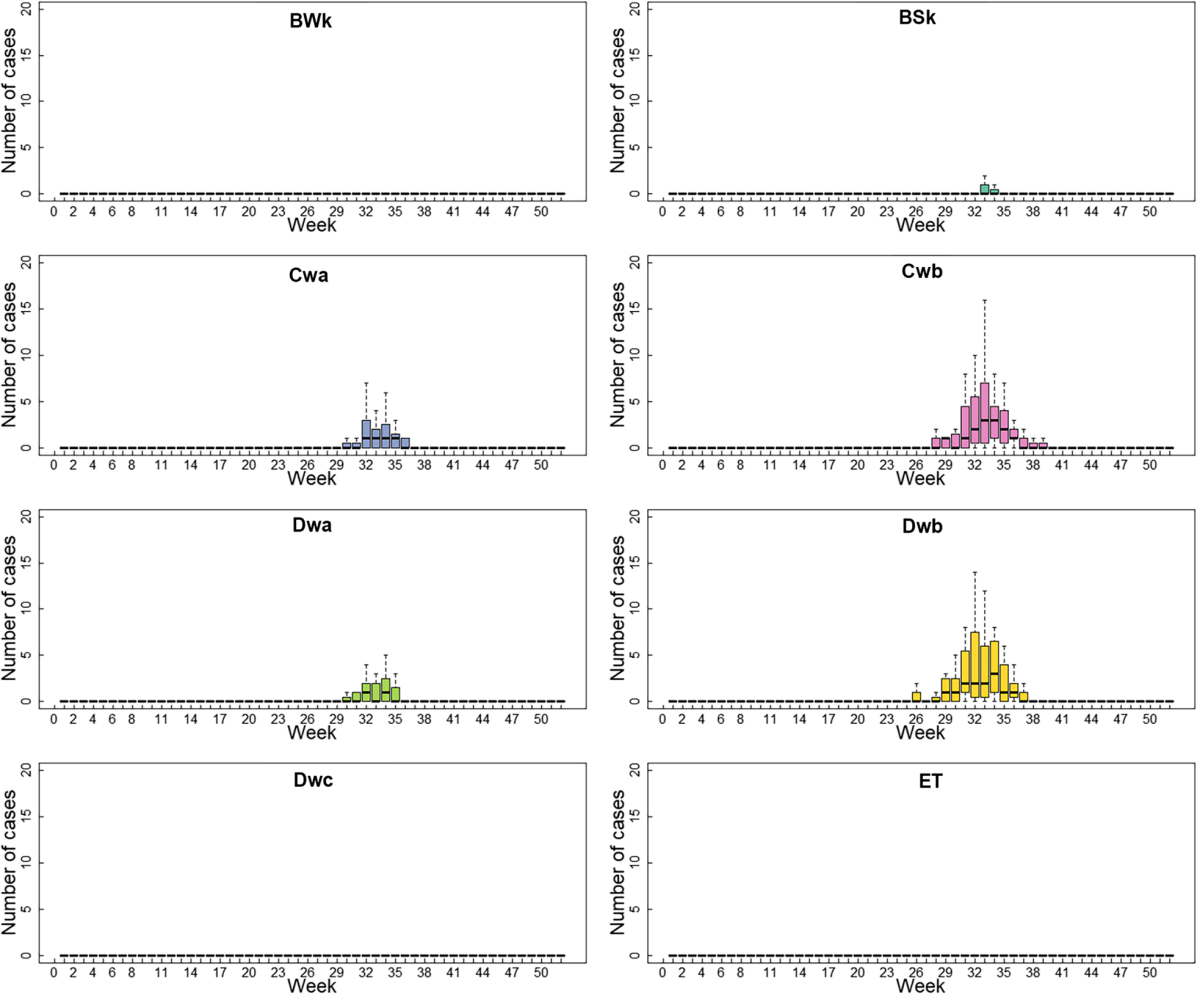
Boxplots of weekly JE cases by climate subtypes in Gansu Province, 2005–2019. The Y-axis indicates the JE cases, the X-axis means a week in a year. Climate types: *BWk* temperate arid, *BSk* temperate semi-arid, *Cwa* subtropical winter dry, *Cwb* temperate oceanic continental, *Dwa and Dwb* continental winter dry, Dwa was characterized by snow climate, dry winter, and hot summer, Dwb was characterized by snow climate, dry winter, and warm summer, *Dwc* subpolar winter dry, *ET* alpine climate

**Fig. 3:**
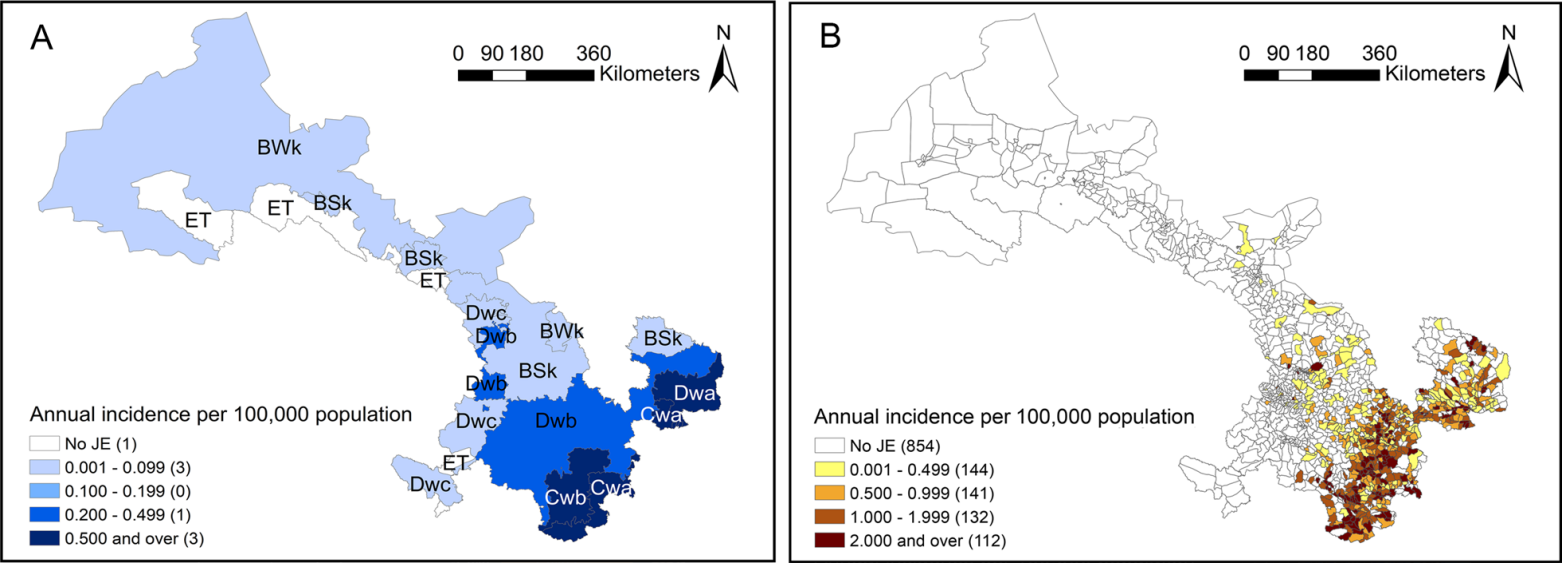
Annual JE incidence rate in Gansu, 2005–2019. **A** Annual JE cases incidence rate (per 100,000 population) in different climate zones in Gansu from 2005 to 2019. **B** The town-level annual JE incidence rate (per 100,000 population) in Gansu. *BWk* temperate arid, *BSk* temperate semi-arid, *Cwa* subtropical winter dry, *Cwb* temperate oceanic continental, *Dwa and Dwb* continental winter dry, Dwa was characterized by snow climate, dry winter, and hot summer, Dwb was characterized by snow climate, dry winter, and warm summer, *Dwc* subpolar winter dry, *ET* alpine climate

Seven climate subtypes in Gansu experienced an epidemic in 2018 (except the ET without JE cases), and this was the first instance of a JE epidemic in some town located in BWk and BSk during the study period. The Cwa, Cwb, and Dwb experienced two JE epidemics in 2006 and 2018, and the Dwa experienced a JE epidemic in 2006, 2012, and 2018 (Fig. 1). The annual JE epidemic period was from the 31st to the 37th week (Fig. 2). The highest mean weekly incidence rate (0.236 per 100,000) was in Cwa, and the highest-peaking weekly incidence rate (1.872 per 100,000) was in Cwb (Table 1). The incidence rate of JE is marked on the map (Fig. 3). The Cwa, Cwb, Dwa, and Dwb had higher incidence rates compare to other four climate subtypes (Table 1).

**Table 1:** Characteristic of mean weekly JE incidence rate by climate zones in Gansu Province from 2005 to 2019

Climate zone	Mean incidence rate in weeks with JE cases	Mean weekly peaking incidence rate
BWk	0.033	0.057
BSk	0.037	0.130
Cwa	0.236	1.517
Cwb	0.205	1.872
Dwa	0.167	0.772
Dwb	0.054	0.549
Dwc	0.098	0.099
ET	0	0.000

*BWk* temperate arid, *BSk* temperate semi-arid, *Cwa* subtropical winter dry, *Cwb* temperate oceanic continental, *Dwa and Dwb* continental winter dry, Dwa was characterized by snow climate, dry winter, and hot summer, Dwb was characterized by snow climate, dry winter, and warm summer, *Dwc* subpolar winter dry, *ET* alpine climate

### Epidemic temporal characteristics analysis

Among the eight climate subtypes, Cwb had the highest frequency index (*α*) at 1.617; Cwa and Dwc had the greatest intensity index (*γ*) at 0.007; and Dwc had the lowest frequency index (*α*) at 0.006 and the lowest duration index (*β*) at 1.000 (Table 2).

**Table 2:** Statistics of epidemic indices of JE in climate zones of Gansu, 2005–2019

Climate zone	Cases	Population	EW	TW	EV	IR	Frequency index (*α*)	Duration index (*β*)	Intensity index (*γ*)
BWk	8	51,455,688	8	783	8	0.016	0.010	1.000	0.002
BSk	66	100,848,086	66	783	63	0.065	0.084	1.048	0.001
Cwa	164	16,033,155	156	783	141	1.023	0.199	1.106	0.007
Cwb	455	28,135,369	403	783	348	1.617	0.515	1.158	0.005
Dwa	147	28,087,413	135	783	121	0.523	0.172	1.116	0.004
Dwb	674	146,717,627	615	783	549	0.459	0.785	1.120	0.001
Dwc	5	15,272,584	5	783	5	0.033	0.006	1.000	0.007
ET	0	2,675,168	0	783	0	0	0	–	–

*BWk* temperate arid, *BSk* temperate semi-arid, *Cwa* subtropical winter dry, *Cwb* temperate oceanic continental, *Dwa and Dwb* continental winter dry, Dwa was characterized by snow climate, dry winter, and hot summer, Dwb was characterized by snow climate, dry winter, and warm summer, *Dwc* subpolar winter dry, *ET* alpine climate, *EW* epidemic weeks, the total number of weeks in which one or more cases are notified, *TW* total weeks, means the total number of weeks during study period, *EV* epidemic waves are defined as the number of weeks when cases successively occur, *IR* incidence rate, the weekly incidence rate (cases per 100,000 population) during a JE epidemic period

### Distribution of JE epidemic trends

**Fig. 4:**
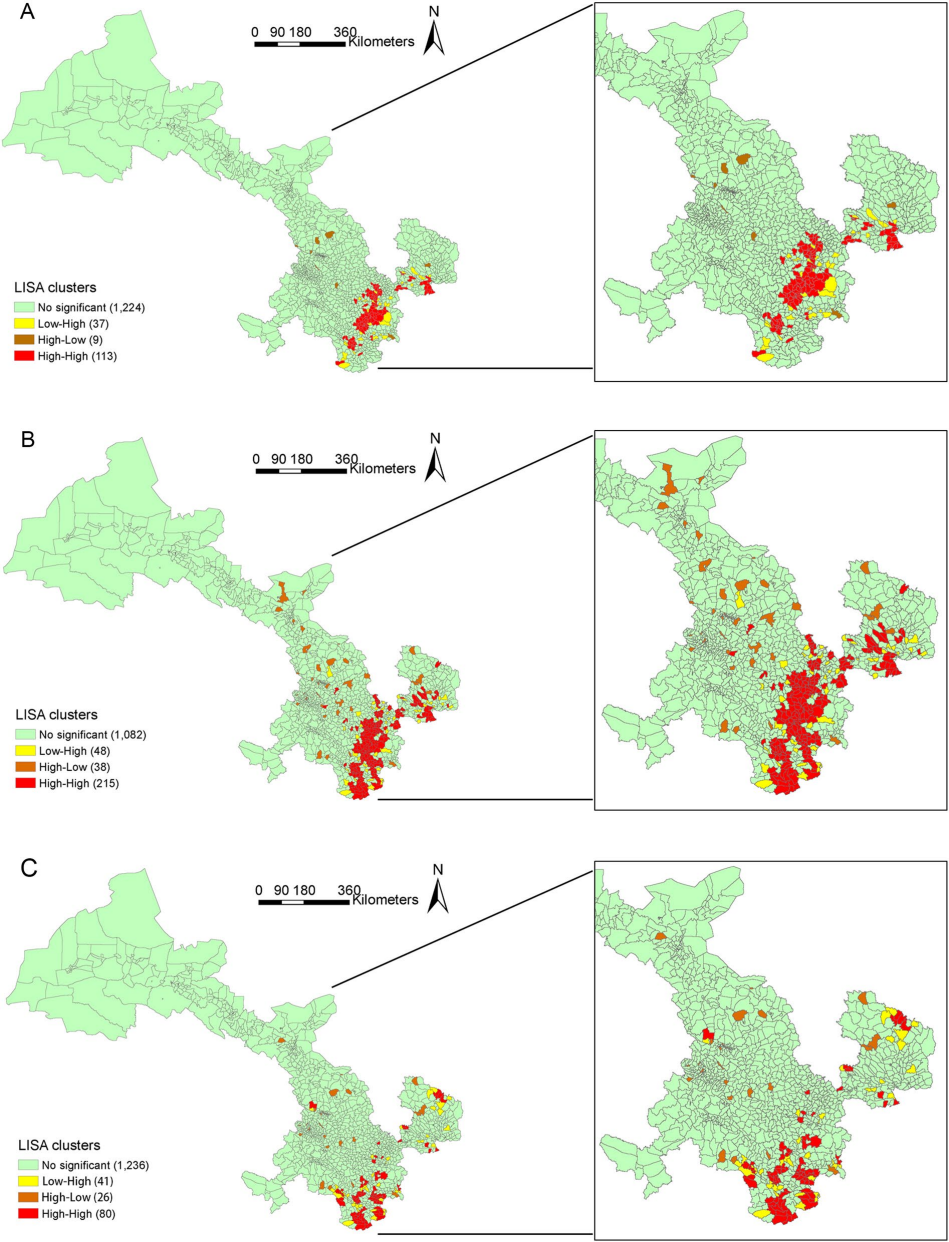
Town-level local spatial autocorrelation analysis of JE in Gansu, 2005–2019. **A** spatial cluster of frequency index (*α*) in Gansu, **B** spatial cluster of duration index (*β*) in Gansu, **C** spatial cluster of intensity index (*γ*) in Gansu. *Low–Low cluster of JE were not exist in Gansu province, and the High–High means hot-spot area

The three epidemic temporal indices had significant positive spatial autocorrelation, and the results of spatial-autocorrelation are presented in Additional file 1: Table S1 (Moran’s *I* in *α*: 0.496, *β*: 0.468, *γ*: 0.264; *p-*value < 0.05). These results showed that low–low cluster did not exist in Gansu Province, and 54 towns in Cwb (126 towns in Cwb) had hot-spot areas of α, which indicated that the α of JE was high in those towns and their surrounding areas. In Dwb, 81 towns (529 towns in Dwb) had hot-spot areas of *β*; and in Cwb, 32 towns (126 towns in Cwb) had hot-spot areas of *γ* (Additional file 1: Table S2, Fig. 4). In addition, epidemic temporal index distribution in the climate subtype presented hot-spot areas, where the high–low or low–high clusters with high–high clusters (hot-spot areas) were detected simultaneously. Hot-spot areas of *α*, *β*, and *γ *were observed in Cwa, Cwb, Dwa, and Dwb.

### Interaction of weather variables and JE cases

The Cwa, Cwb, Dwa, and Dwb were the predominant JE epidemic areas. Therefore, the CARTs were conducted in those areas. The univariate linear regression analysis indicated that the weather variables were significantly positively related to the JE incidence rate (Table 3).

**Table 3:** Univariate linear regression analysis of weather conditions and incidence rate among high-risk climate zones

Variable	Cwa		Cwb		Dwa		Dwb	
	B	*p*-value	B	*p*-value	B	*p*-value	B	*p*-value
Preci (mm)	0.002	< 0.001	0.004	< 0.001	0.002	< 0.001	0.002	< 0.001
preci1 (mm)	0.003	< 0.001	0.005	< 0.001	0.002	< 0.001	0.003	< 0.001
preci2 (mm)	0.001	0.001	0.003	< 0.001	0.001	0.001	0.002	< 0.000
preci3 (mm)	0.001	0.142	0.002	0.004	0	0.38	0.001	0.031
ave.temp (℃)	0.014	< 0.001	0.022	< 0.001	0.007	< 0.001	0.009	< 0.001
ave.temp1 (℃)	0.015	< 0.001	0.025	< 0.001	0.009	< 0.001	0.011	< 0.001
ave.temp2 (℃)	0.013	< 0.001	0.021	< 0.001	0.007	< 0.001	0.009	< 0.001
ave.temp3 (℃)	0.008	0.001	0.013	< 0.001	0.005	0.003	0.006	< 0.001
min.temp (℃)	0.015	< 0.001	0.023	< 0.001	0.008	< 0.001	0.01	< 0.001
min.temp1 (℃)	0.016	< 0.001	0.025	< 0.001	0.009	< 0.001	0.011	< 0.001
min.temp2 (℃)	0.012	< 0.001	0.019	< 0.001	0.007	< 0.001	0.009	< 0.001
min.temp3 (℃)	0.006	0.0015	0.01	0.002	0.004	0.015	0.005	0.001
max.temp (℃)	0.013	< 0.001	0.02	< 0.001	0.006	< 0.001	0.009	< 0.001
max.temp1 (℃)	0.015	< 0.001	0.023	< 0.001	0.008	< 0.001	0.011	< 0.001
max.temp2 (℃)	0.013	< 0.001	0.021	< 0.001	0.007	< 0.001	0.01	< 0.001
max.temp3 (℃)	0.009	< 0.001	0.014	< 0.001	0.005	0.001	0.007	< 0.001

*Preci1* precipitation at the lag of 1 month for JE, *Ave.temp1* the average temperature at the lag of 1 month for JE, *Min.temp1* the minimum temperature at the lag of 1 month for JE, *Max.temp1* the maximum temperature at the lag of 1 month for JE, *Cwa* subtropical winter dry, *Cwb* temperate oceanic continental, *Dwa and Dwb* continental winter dry, Dwa was characterized by snow climate, dry winter, and hot summer, Dwb was characterized by snow climate, dry winter, and warm summer

Weather conditions may influence the JE incidence rate, which varied according to the climate subtypes. The optimum tree for Cwa had four nodes, Cwb had three nodes, and Dwa and Dwb had two terminal nodes. In terms of weather variables, the minimum temperature, average temperature, and precipitation for JE at the lag of 1 month in Cwa, the minimum and average temperature for JE at the lag of 1 month in Cwb, the minimum temperature and average temperature for JE at the lag of 1 month in Dwa, and the minimum and average temperature for JE at the lag of 1 month in Dwb were all found to be predictors of JE incidence during the study period.

The variable at the root node indicates the strongest association among those weather variables. In Cwa, the JE incidence rate increased by approximately 11-fold (to a mean monthly incidence rate of 0.980 per 100,000 compared with an overall mean monthly incidence rate of 0.086 per 100,000 in Cwa), when the monthly average temperature for JE at the lag of 1 month was ≥ 19 °C. The incidence rate decreased to 0.038 per 100,000 when the average temperature for JE at the lag of 1 month was < 19 °C. The incidence rate of JE decreased to 0.008 per 100,000 when the monthly minimum temperature for JE at the lag of 1 month was < 19 °C, and the average temperature for JE at the lag of 1 month was < 20 °C. The incidence rate increased to 0.190 per 100,000 when the monthly minimum temperature for JE at the lag of 1 month was < 19 °C, and the average temperature for JE at the lag of 1 month was ≥ 20 °C. The incidence rate increased to 0.100 per 100,000 when the monthly minimum temperature for JE at the lag of 1 month was < 19 °C, and the average temperature for JE at the lag of 1 month was ≥ 20 °C, and the monthly precipitation for JE at the lag of 1 month was < 107 mm. The incidence rate increased to 0.410 per 100,000 when the monthly minimum temperature for JE at the lag of 1 month was < 19 °C; the monthly average temperature for JE at the lag of 1 month was ≥ 20 °C, and the monthly precipitation for JE at the lag of 1 month was ≥ 107 mm.

In Cwb, the monthly JE incidence rate increased by approximately 14-fold (to a mean monthly incidence rate of 1.800 per 100,000 compared with an overall mean monthly incidence rate of 0.140 per 100,000 in Cwb), when the monthly minimum temperature for JE at the lag of 1 month was ≥ 19 °C. The incidence rate decreased to 0.066 per 100,000 when the monthly minimum temperature for JE at the lag of 1 month was < 19 °C. The incidence rate increased to 0.320 per 100,000 when the monthly minimum temperature for JE at the lag of 1 month was < 19 °C, and the monthly average temperature for JE at the lag of 1 month was ≥ 20 °C. The incidence rate of JE decreased to 0.012 per 100,000 when the monthly minimum temperature for JE at the lag of 1 month was < 19 °C, and the monthly average temperature for JE at the lag of 1 month was < 20 °C.

In Dwa, the monthly incidence rate of JE increased by approximately 18-fold (mean monthly incidence rate of 0.830 per 100,000 compared with an overall mean monthly incidence rate of 0.045 per 100,000 in Dwa) when the minimum temperature for JE at the lag of 1 month was ≥ 18 °C. The monthly incidence rate of JE decreased to 0.013 per 100,000 when the minimum temperature for JE at the lag of 1 month was < 18 °C. The incidence rate of JE increased to 0.001 per 100,000 when the minimum temperature for JE at the lag of 1 month was < 18 °C, and the monthly average temperature for JE at the lag of 1 month was < 20 °C. The incidence rate of JE increased to 0.060 per 100,000 when the minimum temperature for JE at the lag of 1 month was < 18 °C, and the monthly average temperature for JE at the lag of 1 month was < 20 °C.

In Dwb, the JE incidence rate increased by approximately 15-fold (monthly incidence rate of 0.560 per 100,000 compared with an overall mean monthly incidence rate of 0.038 per 100,000 in Dwa) when the minimum temperature for JE at the lag of 1 month was ≥ 16 °C. The monthly incidence rate of JE decreased to 0.017 per 100,000 when the minimum temperature for JE at the lag of 1 month was < 16 °C. The incidence rate of JE decreased to 0.004 per 100,000 when the minimum temperature for JE at the lag of 1 month was < 13 °C. The incidence rate of JE increased to 0.087 per 100,000 when the minimum temperature for JE at the lag of 1 month was ≥ 13 °C and < 16 °C (Additional file 1: Fig. S2).

## Discussion

The Dwc was more likely to experience concentrated JE outbreak because of its low-frequency duration index and its high intensity index. The Cwa, Cwb, Dwa, and Dwb had more hot-spot areas of JE epidemic indices, therefore, those areas were high-risk JE infectious regions in Gansu Province. The weather conditions, especially the monthly minimum temperature and average temperature for JE at the lag of 1 month, may have influenced the JEV transmission. This town-level JE epidemic analysis was conducted based on climate subtypes, and the findings may shed new light on JE management and prevention, particularly in areas where the climate is similar.

The current study found that JE mostly occurred between weeks 31 and 37 of the year. This result was consistent with the studies conducted in provinces neighboring Gansu like Shaanxi and Ningxia, which indicated that JE is prevalent from July to September [14, 31, 32]. A study conducted based on the geographical distribution of JE also found that JE occurs in temperate areas (including Cwa, Cwb, Dwa, and Dwb) in the summer or early autumn [21, 33]. One possible explanation is that JEV is transmitted through a complex cycle between mosquitoes and vertebrate hosts, mainly domestic pigs, and climate influences the development of mosquitoes and viruses as well as the distribution of hosts and vectors. Therefore, JEV transmission is easily influenced by changing climatic conditions.

Further studies are needed to investigate whether climate change contributes to JEV expansion. Some towns in BSk and BWk of northwest Gansu have become new epidemic areas [14]. The emergence of the epidemic represents a new challenge for the prevention and control of JE. Those cases were not travelers from a JE epidemic area. Climate change may contribute to the BWk have longer epidemic duration with fewer cases. A study conducted in BWk climate subtype found positive temperature trends in 1995–2005 [34], and climate change may influence the hydrological cycle [35]. Besides, the BSk owned hot-spot areas of *β* and *γ*. The climate change may contribute to the new epidemic area appear in BSk [36]. While whether this climate change contribute to the JEV transmission in temperate arid (BWk and BSk) is understudied. Results in Dwc require attention. During the study period, before 2018, the Dwc never had a case in Gansu Province. The Dwc had five cases with the highest *γ*, low *α*, and low *β*. The greater *γ* meant a higher incidence rate with lower epidemic waves, which indicated a concentrated onset of infectious diseases [24]. Thus, the Dwc experienced a temporally concentrated JE epidemic in 2018. Moreover, the Dwc is a subarctic climate that borders the Tibetan Plateau [19]. The Tibetan Plateau has been recognized internationally as a nonepidemic area of JE. Because of the high average elevation, it is difficult for the JEV to transmit between mosquitoes and vertebrates [37]. In recent years, however, JEV was detected in Tibet [38]. The possible explanation is the Dwc have fewer population, which makes the Dwc owned higher average incidence rate. A study showed a clear warming trend in China, and areas of the Tibetan Plateau between Ds (Dwb and Dwc) and ET were the main site for these climate changes [39]. Therefore, JEV’s distribution has expanded to Dwc in Gansu Province. More research is needed to investigate whether climate shifts have facilitated the expansion of JEV distribution to Dwc.

The areas in a temperate climate (including Cwa and Cwb) or continental climate (Dwa and Dwb) in Gansu were at high risk of JE incidence. Those areas had more JE cases with the moderate–high epidemic temporal index and showed the most high–high clusters of epidemic temporal indices (*α*, *β*, and *γ*). A previous study also showed that JEV was mainly prevalent in the temperate areas of Asia [40]. Notably, the Cwb was the dominant JE epidemic area in Gansu; compared to other climate zones in Gansu, it has more town-level hot-spots of *α*, *β*, and *γ*. This result may indicate that the Cwb has more favorable climatic conditions or breeding sites that may facilitate JEV transmission. Field studies and laboratory research investigating the water and breeding sites that allow mosquitoes to incubate, as well as the density, vaccine rate, and JE infection rate of pigs, are needed to detect the genotype of JEV in Cwb areas.

Temperature may be the dominant factor that influences the monthly JE incidence in Gansu. In Cwa, Cwb, Dwa, and Dwb, the most predominant predictor of monthly JE incidence was the minimum temperature for JE at a 1-month lag. Another study also demonstrated that temperature was the primary factor that influenced JEV transmission [11]. The temperature threshold of JE response varied in different climate areas, and climate classification may be associated with this outcome. Among the four major climate types (B, C, D, and E) in Gansu, only the areas in the temperate climate (Cwa and Cwb) and continental climate (Dwa and Dwb) exceeded the warmest month’s average temperature of 10 °C [19, 41]. Suitable weather conditions may facilitate JEV transmission. The optimal temperature can decrease the larval development time and reduce the extrinsic incubation period for the virus [11], which further increases the mosquitoes’ density and transmission of the virus. For example, the average temperature ranges from 22.8 to 34.5 °C in India [16], and temperatures ranging from 21.0 to 25.2 °C in China were optimal for JEV transmission [17]. Although other studies have been conducted at the national scale, they have not identified the relationship between JE incidence and weather conditions based on climate subtypes. The current study probed the temperature threshold of JE response based on climate subtypes. These results may help public health administrators develop prevention methods (e.g., vaccinating vertebrates, setting mosquito traps) according to local temperatures.

To the best of our knowledge, this is the first study based on climate subtypes of Gansu to examine the relationship between weather conditions and JE. The CART analysis helps us find out the interactive effects of weather on JE with a further determination of the threshold values of the potential predictors of JE. Specific results of the threshold from CARTs could be used for decision-making, and the lag time associated with the thresholds allows the government to set mosquito traps, vaccinate the hosts against JE, and prepare for the outbreaks in high-risk JE areas. Furthermore, this study could help to identify the efficiency of control measures in JE epidemic areas. However, there are also some limitations to our study. Although this study may interpret the spatial heterogeneity of the JE incidence rate among climate subtypes, it cannot explain why the spatial heterogeneity of JE incidence exists within climate subtypes. In addition, information about vectors and vertebrate density was not available. More studies on etiology (e.g., disease surveillance, vector mosquito surveillance, investigation of the virus load in vectors and hosts (pigs), and antibody levels in healthy people) are essential for the prediction of JEV transmission.

## Conclusions

Further studies are needed to detect whether the JEV transmission has expanded to areas with temperate arid (BWk and BSk) or subpolar winter dry (Dwc) climates. The minimum temperature at a 1-month lag may be the strongest predictor of JE response in subtropical winter dry (Cwa), temperate oceanic continental (Cwb), and continental winter dry (Dwa and Dwb) climates. More in-depth studies on virus and vector biology are needed in areas with temperate oceanic continental (Cwb) climates to investigate predominant JE epidemic areas.

## Supplementary Information

**Additional file 1: Table S1.** Global spatial autocorrelation analysis results of JE in climate zones among Gansu Province, 2005–2019. **Table S2.** Town-level local spatial autocorrelation analysis results of JE in climate zones of Gansu Province, 2005–2019. **Figure S1.** The climate stations in different climate zone, Gansu, China. **Figure S2.** Regression tree modeling the hierarchical relationship between JE incidence rate and weather variables in four different climate zones of Gansu, China during 2005–2019.

## Data Availability

The datasets analyzed in the current study are not publicly available due to protection of privacy but are available from the corresponding author upon reasonable request.
