# Supplementary material for: Different responses of Japanese encephalitis to weather variables among eight climate subtypes in Gansu, China, 2005–2019
Source: BMC Infect Dis. 2023 Feb 23;23:114. doi: 10.1186/s12879-023-08074-6 (PMC9951518; doi:10.1186/s12879-023-08074-6)
Supplement: Supplementary file 1 — Additional file 1: Table S1. Global spatial autocorrelation analysis results of JE in climate zones among Gansu Province, 2005–2019. Table S2. Town-level local spatial autocorrelation analysis results of JE in climate zones of Gansu Province, 2005–2019. Figure S1. The climate stations in different climate zone, Gansu, China. Figure S2. Regression tree modeling the hierarchical relationship between JE incidence rate and weather variables in four different climate zones of Gansu, China during 2005–2019. [file 12879_2023_8074_MOESM1_ESM.docx]

Table S1 Global spatial autocorrelation analysis results of JE in climate zones among Gansu Province, 2005-2019.

| Epidemic Temporal indices | Moran'*I* | E(I) | Mean | Sd | Z-value | *P*-value |
| --- | --- | --- | --- | --- | --- | --- |
| Frequency index (α) | 0.496 | -0.001 | -0.002 | 0.018 | 27.816 | <0.001 |
| Duration index (*β*) | 0.468 | -0.001 | -0.001 | 0.018 | 25.830 | <0.001 |
| Intensity index (*γ*) | 0.264 | -0.001 | -0.002 | 0.018 | 15.082 | <0.001 |

Table S2 Town-level local spatial autocorrelation analysis results of JE in climate zones of Gansu Province, 2005-2019.

|  |  | Frequency index(α) | | | |  | Duration index(*β*) | | | |  | Intensity index(*γ*) | | | |
| --- | --- | --- | --- | --- | --- | --- | --- | --- | --- | --- | --- | --- | --- | --- | --- |
| Climate Zone | Number of towns | No significant | L-H^*^ | H-L^**^ | H-H^***^ | | No significant | L-H | H-L | H-H | | No significant | L-H | H-L | H-H |
| BWk | 193 | 193 | 0 | 0 | 0 |  | 188 | 0 | 5 | 0 |  | 190 | 0 | 3 | 0 |
| BSk | 265 | 261 | 0 | 4 | 0 |  | 248 | 1 | 14 | 2 |  | 250 | 3 | 8 | 4 |
| Cwa | 88 | 74 | 9 | 1 | 4 |  | 39 | 10 | 1 | 38 |  | 47 | 13 | 1 | 27 |
| Cwb | 126 | 63 | 9 | 0 | 54 |  | 42 | 8 | 1 | 75 |  | 83 | 10 | 1 | 32 |
| Dwa | 80 | 69 | 2 | 1 | 8 |  | 53 | 7 | 1 | 19 |  | 77 | 2 | 0 | 1 |
| Dwb | 529 | 462 | 17 | 3 | 47 |  | 412 | 22 | 14 | 81 |  | 488 | 13 | 12 | 16 |
| Dwc | 86 | 86 | 0 | 0 | 0 |  | 84 | 0 | 2 | 0 |  | 85 | 0 | 1 | 0 |
| ET | 16 | 16 | 0 | 0 | 0 |  | 16 | 0 | 0 | 0 |  | 16 | 0 | 0 | 0 |
| Total | 1383 | 1224 | 37 | 9 | 113 |  | 1082 | 48 | 38 | 215 |  | 1236 | 41 | 26 | 80 |

*L-H: Low-High cluster;

**H-L: High-Low cluster;

***H-H: High-High cluster (hot-spot area), the Low-Low cluster of JE were not exist in the current study.

BWk: temperate arid,

BSk: temperate semi-arid,

Cwa: subtropical winter dry,

Cwb: temperate oceanic continental,

Dwa and Dwb: continental winter dry, Dwa was characterized by Snow climate, dry winter, and hot summer, Dwb was characterized by Snow climate, dry winter, and warm summer.

Dwc: subpolar winter dry,

ET: alpine climate.


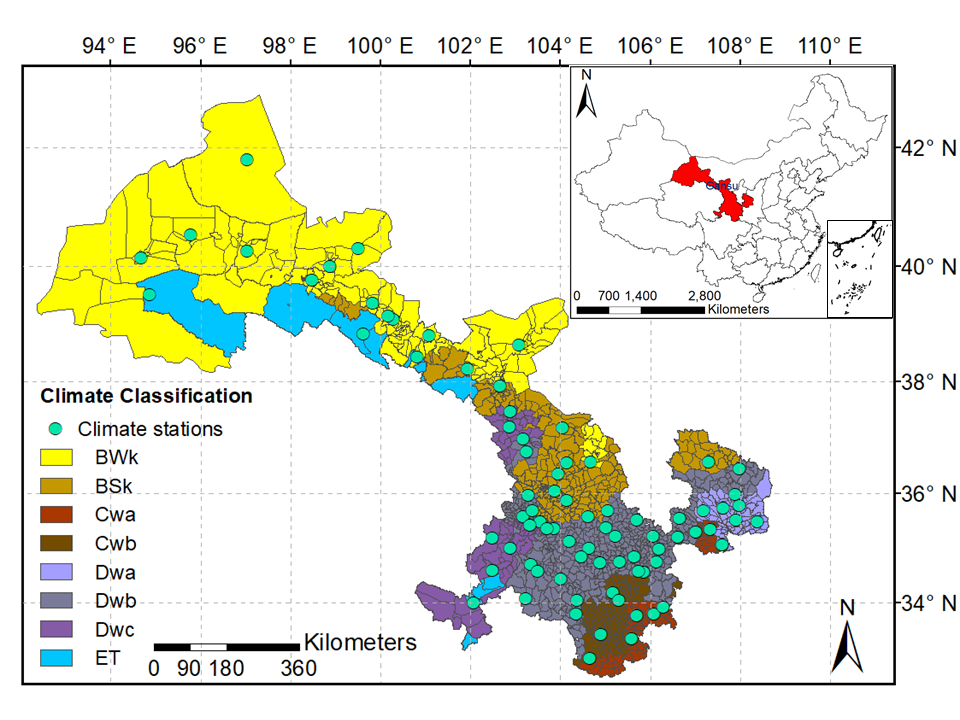


**Figure S1 The climate stations in different climate zone, Gansu, China.**

Cwa: subtropical winter dry,

Cwb: temperate oceanic continental,

Dwa and Dwb: continental winter dry, Dwa was characterized by snow climate, dry winter, and hot summer, Dwb was characterized by snow climate, dry winter, and warm summer


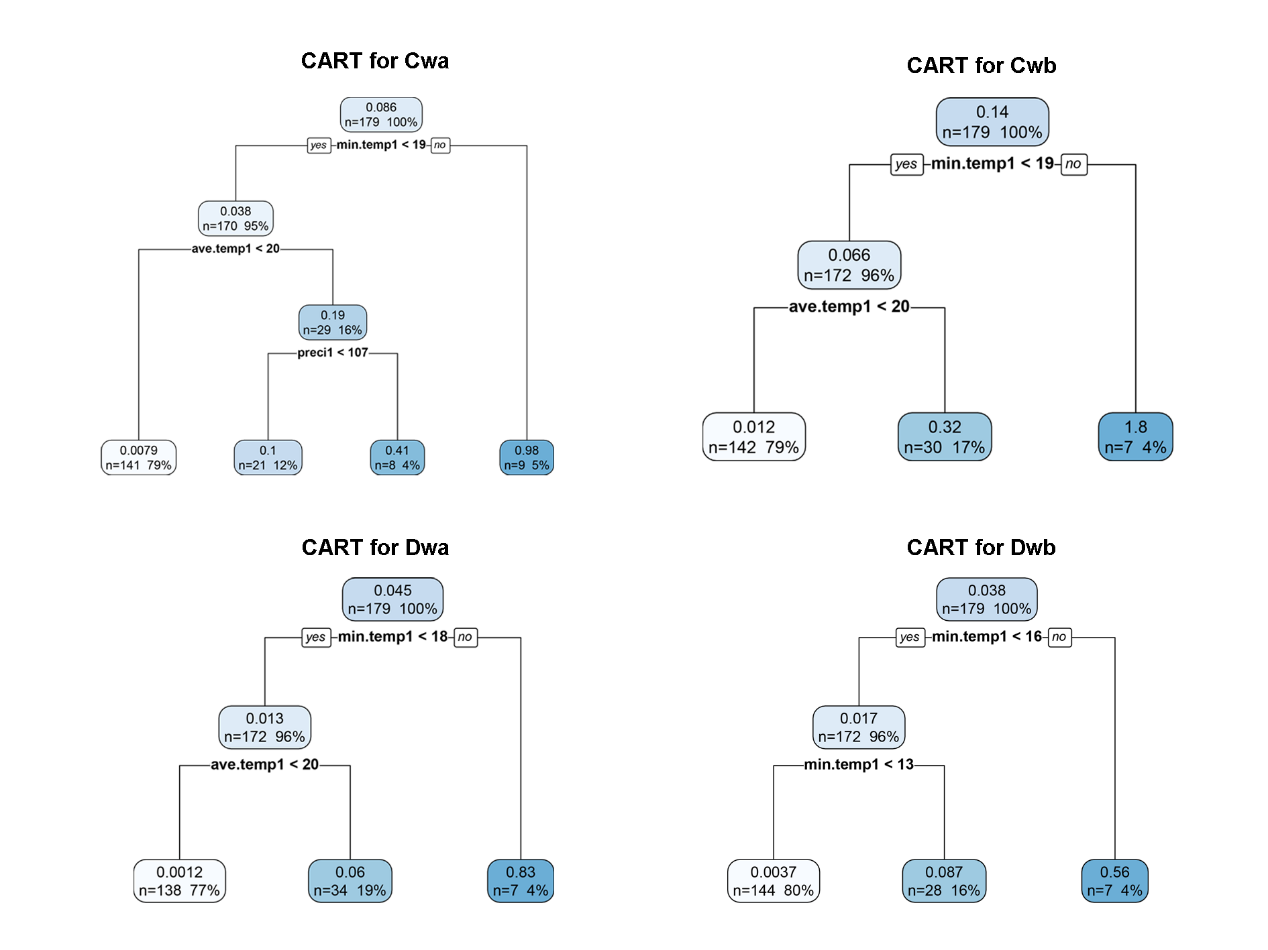


Figure S2 Regression tree modeling the hierarchical relationship between JE incidence rate and weather variables in four different climate zones of Gansu, China during 2005-2019. Ave.temp1: the average temperature at the lag of 1 month for JE; Max.temp1: the maximum temperature at the lag of 1 month for JE; Min.temp1: the minimum temperature at the lag of 1 month for JE, n: the number of months had the expected incidence rate. Every node has the expected incidence rate and the percentage rate means the probability of the incidence rate.

Cwa: subtropical winter dry,

Cwb: temperate oceanic continental,

Dwa and Dwb: continental winter dry, Dwa was characterized by snow climate, dry winter, and hot summer, Dwb was characterized by snow climate, dry winter, and warm summer

Dwc: subpolar winter dry.
